# A Multicenter Retrospective Review of Prone Position Ventilation (PPV) in Treatment of Severe Human H7N9 Avian Flu

**DOI:** 10.1371/journal.pone.0136520

**Published:** 2015-08-28

**Authors:** Yuanda Xu, Xilong Deng, Yun Han, Lixin Zhou, Weiqun He, Sibei Chen, Lingbo Nong, Huang Huang, Yan Zhang, Tieou Yu, Yimin Li, Xiaoqing Liu

**Affiliations:** 1 State Key Laboratory of Respiratory Diseases, Department of Critical Care Medicine, First Affiliated Hospital of Guangzhou Medical University, Guangzhou, 510120, China; 2 Department of Critical Care Medicine, Guangzhou Eighth People’s Hospital, Guangzhou, 510060, China; 3 Department of Critical Care Medicine, Fangcun Branch of Guangdong Hospital of Traditional Chinese Medicine, Guangzhou, 510360, China; 4 Department of Critical Care Medicine, Foshan First People’s Hospital, Foshan, 528000, China; Zhongshan Hospital Fudan University, CHINA

## Abstract

**Background:**

Patients with H7N9 avian flu concurrent with severe acute respiratory distress syndrome (ARDS) usually have a poor clinical outcome. Prone position ventilation (PPV) has been shown to improve the prognosis of patients with severe ARDS. This study explored the effects of PPV on the respiratory and circulatory mechanics of H7N9-infected patients with severe ARDS.

**Methods:**

Individuals admitted to four hospitals designated for H7N9 patients in Guangdong province were treated with PPV, and their clinical data were recorded before and after receiving PPV.

**Results:**

Six of 20 critically ill patients in the ICU received PPV. After treatment with 35 PPV sessions, the oxygenation index (OI) values of the six patients when measured post-PPV and post-supine position ventilation (SPV) were significantly higher than those measured pre-PPV (P < 0.05).The six patients showed no significant differences in their values for respiratory rate (RR), peak inspiratory pressure (PIP), tidal volume (TV) or arterial partial pressure of carbon dioxide (PaCO_2_) when compared pre-PPV, post-PPV, and post-SPV. Additionally, there were no significant differences in the mean values for arterial pressure (MAP), cardiac index (CI), central venous pressure (CVP), heart rate (HR), lactic acid (LAC) levels or the doses of norepinephrine (NE) administered when compared pre-PPV, post-PPV, and post-SPV.

**Conclusion:**

PPV provided improved oxygenation that was sustained after returning to a supine position, and resulted in decreased carbon dioxide retention. PPV can thus serve as an alternative lung protective ventilation strategy for use in patients with H7N9 avian flu concurrent with severe ARDS.

## Introduction

Following its previous spread throughout Eastern China, human cases of H7N9 avian flu were reported in Guangdong Province during the winter of 2013. Because of its persistent presence in chickens, H7N9 influenza virus may become a long-term threat to public health [1]. Statistics provided by the Guangdong Center for Disease Control and Prevention (CDC) indicated that from January, 2014 to mid-April, 2014, there were 103 confirmed cases and 33 fatal cases of H7N9 avian flu. Among the 103 confirmed cases, ~ 70% of the patients became critically ill. In addition to fever and cough, avian flu can manifest with rapidly progressive severe pneumonia and severe acute respiratory distress syndrome (ARDS). In 2014, the Chinese Ministry of Health (MOH) developed policies and procedures designed to protect against H7N9 virus infection (2014 Guidelines for Prevention and Control of Human H7N9 Avian Flu) that recommended the use of both protective prone position ventilation (PPV) and supine position ventilation (SPV) when treating H7N9 infected patients with respiratory dysfunction. Hence, a protective PPV strategy has been utilized with some patients receiving antiviral therapy, and who exhibited refractory hypoxemia and persistent pulmonary exudation. However, very few studies have evaluated the effect of PPV on the outcome of patients with severe ARDS accompanied by an avian flu infection. Our current multicenter retrospective study was conducted to examine the efficacy achieved when using PPV in a small population of H7N9 avian flu patients in Guangdong Province, China.

## Patients and Methods

The protocol for this retrospective study was reviewed and approved by the Medical Ethics Committee of the First Affiliated Hospital of Guangzhou Medical University (2014 approval number 42). After reading a study information leaflet, a signed written Informed Consent document was obtained from each patient’s next of kin. All patient information was protected for anonymity, and identification markings were removed from patient samples prior to analysis. All data used in this study were collected during routine measurements rather than measurements specifically conducted to support this investigation.

All enrolled subjects were required to have refractory hypoxemia and stable hemodynamics. Subjects with barotrauma or severe cardiac arrhythmia were excluded from enrollment. Based on these criteria, six patients with a documented diagnosis of H7N9 avian flu, and who were subsequently admitted to the negative-pressure isolation ward in the intensive care unit (ICU) from December 2013 to March 2014 were included in this study. All patients satisfied the MOH diagnostic criteria for both human H7N9 avian flu [2] and severe ARDS (2012 Berlin criteria) [3]. According to the “Six–step Standard Management Strategy for ARDS,” extracorporeal membrane oxygenation (ECMO) is the treatment of last resort for ARDS patients who show no improvement of refractory hypoxemia following normal treatment, while PPV is proposed as a secondary treatment. Thus, PPV was provided only after unanimous agreement by the authors of this manuscript [4]. Additionally, PPV was administered as a part of routine medical practice, rather than specifically to assess its efficacy in this study. At the first PPV session, the patients had a mean oxygenation index (OI) of 69.85 ± 14.43, and a protective mechanical ventilation strategy was employed to maintain small tidal volumes, controlled respiratory plateau pressure, and optimal end-expiratory positive pressure (PEEP). The six enrolled patients underwent treatment at the following four hospitals designated for H7N9 patients in Guangdong province: First Affiliated Hospital of Guangzhou Medical University, Guangzhou Eighth People’s Hospital, Fangcun Branch of Guangdong Hospital of Traditional Chinese Medicine, and Foshan First People’s Hospital. All procedures conducted in this study complied with good ethical medical standards, and the study protocol was approved the Ethical Review Board of each hospital.

### PPV conduction

When providing PPV, three or four medical staff members assisted to place the patient in the prone position. Next, the patient’s head was turned to one side to avoid any compression-associated facial injury, and both arms were lifted straight above the head. A soft pillow was then placed under both shoulders and pelvis to prevent abdominal compression and any resultant adverse effects on venous return. The endotracheal tubes, ventilator channels, venous catheters, and other drainage tubes were checked for adequate patency and kept in place throughout the ventilation process. Doses of sedatives and muscle relaxants were adjusted to achieve an ideal depth of sedation, while also maintaining synchrony between the ventilator and the patient’s breathing, as well as adequate circulatory perfusion. During PPV, each patient was routinely examined for evidence of pressure ulcers and endotracheal tube dislodgement, and the eyeballs and conjunctiva were monitored for signs of abnormalities. All patient ventilation procedures were conducted in accordance with Chinese Medical Association Critical Care Medicine Society Guidelines for the Diagnosis and Treatment of ARDS [5].

### Data collection

One full-time physician and one responsible attending physician simultaneously recorded all patient data in a table specifically designed for the study. Data concerning respiratory and circulatory mechanical functions were retrospectively recorded every hour before and after each PPV session. The data recorded for respiratory mechanics included each patient’s dynamic compliance of the respiratory system (Crs), arterial partial pressure of carbon dioxide (PaCO_2_), tidal volume (TV), minute ventilation (MV), respiratory rate (RR), peak inspiratory pressure (PIP), PEEP, arterial blood pH, and OI. The data recorded for circulatory mechanics included readings for cardiac index (CI), central venous pressure (CVP), heart rate (HR), mean arterial pressure (MAP), norepinephrine (NE), and lactic acid (LAC). Each patient’s urine volume (UO) and biochemical parameters including creatinine and urea nitrogen levels were recorded every hour.

### Statistical analysis

All data analyses were performed using IBM SPSS Statistics for Windows, Version 19.0. Armonk, NY: IBM Corp. Results from analyses of quantitative variables are presented as the mean ± standard deviation (SD), if not otherwise defined. Tests for the normality of data distribution and homogeneity of variance were performed prior to conducting intergroup comparisons. When the data showed a normal distribution and an equal variance, randomized block ANOVA was used for comparing data obtained pre-PPV, post-PPV, and post-SPV. The rank sum test was used for comparing data with a non-normal distribution or unequal variance. The Bonferroni method was used for pairwise comparisons. Repeated-measure ANOVA was used to compare changes in the values for clinical parameters at different time points during PPV therapy. A *P*-value < 0.05 was considered statistically significant.

## Results

### General data

Between December 2013 and March 2014, a total of 28 patients admitted to four hospitals were confirmed as H7N9 infection, and 20 patients in ICUs were identified as critically ill patients based on 2014 Guidelines for Prevention and Control of Human H7N9 Avian Flu. Based on the study inclusion criteria, six patients (3 males and 3 females; median age 62 years, range 48–70 years; Table 1) were finally enrolled in the study. The majority of patients had an underlying illness, and all 6 patients had a diffuse pulmonary exudation at the time of hospital admission. Three patients had a confirmed history of contact with poultry. Four patients died during the study period and 2 patients survived. The patients who died had multiple serious complications of their disease. One patient who died of septic shock had shown significantly improved pulmonary ventilation and oxygenation with treatment. The median time from onset of symptoms to initiation of antiviral therapy with oseltamivir was 4 days; range 0 to 10 days (Table 1). Antiviral treatment was initiated earlier in patients who survived for 2 and 4 days, respectively. The median patient score on the acute physiology and chronic health evaluation (APACHE II) administered at the time of hospital admission was 23.5; range 15–36 (Table 1). Patients who survived had scores in the lower range. The six patients underwent a total of 35 PPV sessions, which had a mean duration of 12.86 ± 4.26 h per session; range 5 to 22 h per session (mean the median time from onset of clinical symptoms to initiation of PPV was 9 days; range, 4–16 days). The median duration of patients being positive for viral nucleic acid was 14 days; range 1 to 17 days, and the median duration of therapy with oseltamivir was 11 days; range 5 to 16 days. Additionally, 70% of the patients received combination therapy with two different antiviral agents (Table 1).

**Table 1:** General data for all 6 patients.

Characteristic	H7N9-infected patients
Age, (years)	62 (48–70)
Male sex. No. (%)	3 (50)
Blood type, No. (%)	
A	2 (33.3)
B	1 (16.7)
O	3 (50)
Occupation, No. (%)	
Self-employed	3 (50)
Retired	3 (50)
Underlying illness, No. (%)	
Previously healthy	2 (33.3)
Hypertension	2 (33.3)
Diabetes	1 (16.7)
Renal failure	1 (16.7)
Gastrointestinal disease	2 (33.3)
Exposure to live poultry, No. (%)	3 (50)
Time between onset of symptoms and initiation of oseltamivir. (days)	4 (0–10)
Initial affected lobe on X-ray, No. (%)	
Diffuse	5 (83.3)
Local	1 (16.7)
APACHE II (admission days)	23.5 (15–36)
Complications, No. (%)	
Septic shock	5 (83.3)
DIC	4 (66.7)
Acute kidney injury	4 (66.7)
Hepatic injury	1 (16.7)
Cardiac impairment,	4 (66.7)
MODS	4 (66.7)
Secondary pulmonary infection	6 (100)
Gastrointestinal bleeding	3 (50)
Antibiotics given, No. (%)	
Vancomycin	5 (83.3)
Piperacillin tazobactam	5 (83.3)
Tienam	4 (66.7)
Cefoperazone sulbactam	3 (50)
Meropenem	2 (33.3)
Moxifloxacin	2 (33.3)
Linezolid	2 (33.3)
Levofloxacin	2 (33.3)
Days of hospital stay	39 (4–95)
Time from onset of symptoms to initiation of PPV, (days)	9 (4–16)
Number of sessions of PPV	3.5 (1–19)
Duration of PPV, (hours)	40.5 (13–277)
Outcome, No. (%)	
Survived	2 (33.3)
Fatal	4 (66.7)

Note: Data are presented as the median value (minimum-maximum), unless otherwise indicated.

### Changes in respiratory mechanics

**Fig 1:**
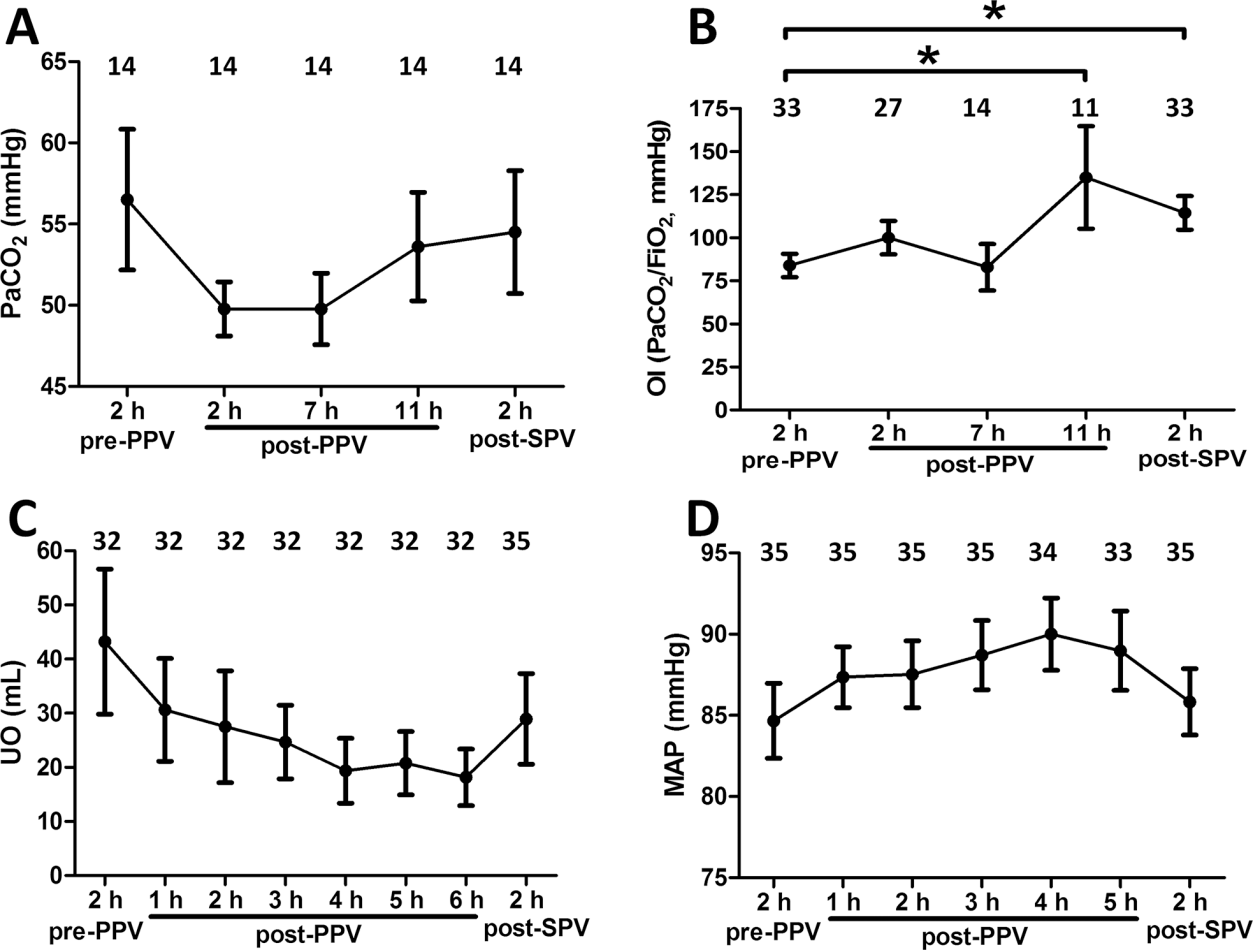
Changes in arterial partial pressure of carbon dioxide (PaCO_2_) (A); oxygenation index (OI, PaO_2_/FiO_2_) (B); urine volume (UO) (C); MAP (D) in H7N9-infected patients with severe acute respiratory distress syndrome (ARDS) at different time points during prone position ventilation (PPV) sessions. The numbers in the upper region of each image represent the number of PPV sessions at different time points. Data are presented as the mean ± SEM, *p < 0.05.

The values for OI (PaO_2_/FiO_2_) recorded post-PPV were significantly greater than those recorded pre-PPV (104.17 ± 55.91 vs. 85.55 ± 41.35; P < 0.05, Table 2). These improvements in OI were sustained post-SPV, and remained significantly different compared to the values pre-PPV (98.69 ± 57.25 vs. 85.55 ± 41.35; P < 0.05, Table 2). There were no significant differences in the values for RR, PEEP, pH, PIP, VT or MV as determined pre-PPV, post-PPV, and post-SPV (P > 0.05, Table 2). Values for PaCO_2_ showed a decrease post-PPV, but increased at post-SPV compared to the values at pre-PPV (P > 0.05, Fig 1A). Values for Crs decreased post-PPV compared to those recorded pre-PPV (P > 0.05), and reflected limited movement of the thoracic cage.

**Table 2:** Changes in parameters of respiratory mechanics during the PPV sessions (from 2 hours pre-PPV to 2 hours post-SPV) (Mean ± SD).

Respiratory mechanics measures	Pre-PPV	Post-PPV	Post-SPV
RR (beats/min)	21.63 ± 2.96	21.77 ± 2.89	21.40 ± 3.12
MV(L)	7.33 ± 2.12	7.52 ± 2.33	7.54 ± 2.38
Crs (mL/cmH_2_O)	21.16 ± 14.61	18.67 ± 7.65	18.32 ± 7.32
OI (PaO_2_/FiO_2_, mmHg)	85.55 ± 41.35	104.17 ± 55.91*	98.69 ± 57.25*
PaCO_2_ (mmHg)	53.85 ± 13.79	51.65 ± 10.90	53.32 ± 11.90
PEEP (cmH_2_O)	13.94 ± 4.61	13.71 ± 4.92	13.89 ± 5.08
pH	7.35 ± 0.07	7.35 ± 0.08	7.34 ± 0.07
PIP (cmH_2_O)	31.06 ± 3.19	32.14 ± 3.29	31.83 ± 3.30
VT (mL)	369.51 ± 153.53	366.72 ± 147.26	369.09 ± 141.51

* P < 0.05 for comparisons of PaO_2_/FiO_2_ post-PPV and post-SPV vs. pre-PPV.

Note: The data presented in this table are mean values. Thus, pre-PPV values represent the mean values of 2-hour results obtained prior to PPV treatment, post-PPV data are the mean values of results obtained after PPV treatment, and post-SPV data are the mean values of 2-hour results obtained after SPV treatment.

### Changes in circulatory mechanics

There were no significant changes in the values for all parameters of circulatory mechanics (CI, CVP, HR, MAP, NE, and LAC) when measured pre-PPV, post-PPV, and post-SPV (P > 0.05, Table 3).

**Table 3:** Changes in parameters of circulatory mechanics during the PPV sessions (from 2 hours pre-PPV to 2 hours post-SPV) (Mean ± SD).

Circulatory mechanics measures	Pre-PPV	Post-PPV	Post-SPV
CI (L/min/m^2^)	3.45 ± 0.95	3.37 ± 0.89	3.45 ± 1.02
CVP (cmH_2_O)	13.48 ± 3.61	13.14 ± 3.80	12.88 ± 3.40
HR (beats/min)	107.05 ± 15.81	105.79 ± 15.43	108.06 ± 17.49
MAP (mmHg)	84.60 ± 10.47	86.45 ± 9.87	85.83 ± 12.08
NE (μg/Kg/min)	0.13 ± 0.19	0.15 ± 0.29	0.22 ± 0.38
LAC (mmol/L)	2.94 ± 1.70	2.35 ± 1.54	3.30 ± 2.15

Note: PICCO was monitored in 3 of 6 patients. The data presented in this table are mean values. Thus, pre-PPV values represent the mean values of 2-hour results obtained prior to PPV treatment, post-PPV data are the mean values of results obtained after PPV treatment, and post-SPV data are the mean values of 2-hour results obtained after SPV treatment.

CI, cardiac index; CVP, central venous pressure; HR, heart rate; MAP, mean arterial pressure; NE, norepinephrine; LAC, lactic acid

### PaCO_2_, OI, urine volume, and MAP before and after PPV

Values for PaCO_2_at 2 h and 7 h post-PPV were lower than those recorded at 2 h pre-PPV; however, the differences were not statistically significant (P > 0.05). Moreover, these lower values for PaCO_2_ gradually increased again over time (Fig 1A). Values for OI significantly increased at 2 h post-PPV compared to those at 2 h pre-PPV (P < 0.05), but then showed a transient decrease at 7 h post-PPV. Significantly higher values for OI were seen at 11 h post-PPV and 2 h post-SPV compared to those at 2 h pre-PPV (P < 0.05) (Fig 1B). The urine volumes recorded between 1 h and 6 h post-PPV were less than the volume recorded 2 h pre- PPV (P > 0.05), but increased again starting at 2 h post-SPV (Fig 1C). Values for MAP were greater at 2 h, 3 h, 4 h, and 5 h post-PPV compared to that recorded 2 h pre-PPV (P < 0.05), which may have be related to a slightly increased dose of NE (P > 0.05) (Fig 1D).

## Discussion

Gao et al. [6] analyzed the clinical manifestations of H7N9 avian flu in 111 patients, and found that most patients displayed serious respiratory symptoms and progressed to pneumonia. Moreover, some cases showed a rapid progression to severe pneumonia and ARDS. Severe ARDS is one of the most common complications of severe human H7N9 avian flu. The H7N9 flu patients in our study group had been positive for viral nucleic acid for a relatively long period time (median positive duration 14 days; range, 1–17 days). They had also received antiviral therapy for a mean duration of 11.8 ± 4.0 days, which is considered a prolonged treatment period compared to those used in treating patients with severe influenza A H1N1 and severe SARS [7, 8]. Seventy percent of our patient cohort received combination therapy with two antiviral agents (e.g., peramivir and zanamivir). In humans, the H7N9 virus tends to reside in the lower respiratory tract, resulting in lung injuries associated with persistent inflammation [9]. The presence of such injuries also suggested the development drug resistance during a course of antiviral therapy [10]. Patients critically ill with ARDS and having refractory hypoxemia and a high ventilator-support-index will develop ventilator-associated lung injuries (VILIs) induced by prolonged ventilator treatment. Thus it is very important to find a method for protecting the lungs of such patients.

Although PPV has long been used as an adjuvant approach for treating ARDS, the idea of using PPV to improve patient ventilation and oxygenation was first proposed in the 1970s [11]. In addition to benefitting alveolar recruitment, preventing pulmonary edema, improving pulmonary homogeneity, and relieving ventilator induced lung injury (VILI), PPV also stimulates secretion drainage and improves the prognosis of patients with severe ARDS[12, 13]. Additionally, it probably does not increase the likelihood of a patient developing ventilator-associated pneumonia (VAP). All patients in our study cohort satisfied the 2012 Berlin diagnostic criteria for severe ARDS [3], which include having an OI of 69.85 ± 14.43 mmHg at initiation of the first PPV session. A patient should not receive PPV if their hemodynamic parameters remain unstable following fluid resuscitation, or if a severe arrhythmia and/or barotrauma is diagnosed. Without ECMO treatment, it is difficult for a patient to recover from refractory hypoxemia and continue to survive with sustained tissue hypoxia. In our current study, all six patients had stable circulation, and thus PPV was the preferred treatment for severe ARDS. Our retrospective data analysis showed significantly higher values for OI post-PPV compared to values pre-PPV. Additionally, there were sustained improvements in patient OI post-SPV compared to post-PPV that were considered related to the persistent improvement in pulmonary homogeneity [14]. The changes in OI were vital for improving the patient’s prognosis following a return to the supine position. In addition to improved oxygenation, dynamic compliance of the respiratory system (Crs) decreased post-PPV compared to pre-PPV. This result most likely occurred because total compliance of the thoracic cage decreased in the prone position due to compression of the frontal thoracic cage [15]. Although dynamic compliance decreased during the PPV therapy, the oxygenation of patients significant improved and carbon dioxide retention released in somehow, which implied prone positioning relieved pressure on the posterior thoracic cage and lung tissue. This effect was more pronounced in patients with clinically significant pulmonary edema in the posterior thoracic area and signs of alveolar collapse. In these patients, PPV produced a more uniformly distributed ventilation/perfusion ratio despite the presence of thoracic cage and lung tissue compression in the lateral abdominal region [16, 17]. Dynamic observations showed a transient decline in OI at 7 h post-PPV; however, the OI values later increased in the absence of increases in PaCO_2_. This finding may result from an excessive positive fluid balance related to continuously decreased urine volumes due to abdominal compression in the prone position. However, after providing symptomatic treatment and enhanced drainage, the OI values showed dramatic increases. According to the report by Guerin C et al. [12, 18], the usual amount of time required for a PPV session in this study was 16 hours, but varied between 5 and 22 hours, based on whether the patient showed decreased oxygenation and increased carbon dioxide levels, shortness of breath, and a high heart rate, which needed to be checked using SPV. Thus, it should be emphasized that to obtain the beneficial effects of PPV, all organ functions should be well coordinated, and dynamic monitoring of pulmonary homogeneity should be performed whenever possible. If these conditions are satisfied, PPV is a good choice for providing lung protection to patients with severe human H7N9 avian flu accompanied by severe ARDS.

ECMO is used for treating ARDS when a patient’s blood oxygen levels fail to significantly improve or a medical condition worsens after appropriate measures had been taken. Such measures include reducing the TV, restricting peak airway pressure, optimizing PEEP, fluid resuscitation, and improving synchrony between a ventilator and the patient’s breathing by providing adequate sedation in accordance with 2012 SSC guidelines. Additionally, in certain phases of patient treatment, PPV is more economical than ECMO and has lower rates of complications. In this study, one patient was switched to ECMO due to invalid PPV therapy, and other patients were switched to ECMO after detection of biotrauma at the early stage of PPV, in spite of having stable circulation.

Our results showed increased MAP readings and NE doses post-PPV compared to those measured at pre-PPV. Additionally, slight improvements in lactic acid levels were seen following PPV; however, the changes were not statistically significant. Each patient’s sedation depth was adjusted to ensure synchrony between the ventilator and the patient’s breathing; and under such conditions, the CI levels remained stable so long as significant changes were not made to ensure adequate oxygen perfusion of vital organs such as the heart, brain, and kidneys. The significantly decreased urine volumes observed post-PPV may be related to decreased blood flow due to compression of kidney blood vessels resulting from abdominal compression. Hering et al. [13] reported that slight increases in intra-abdominal pressure following PPV had no affect on renal blood flow, however large increases in pressure affected both renal perfusion and function. Septic shock, acute kidney injury (AKI), and abdominal bloating are common occurrences in patients with severe pneumonia concurrent with ARDS, and four of the six patients in our study cohort developed AKI. The decreased urine volumes in the presence of a stable circulatory mechanism indicated the need to avoid significant compression of the abdomen during PPV. When taking this precaution, the possible adverse effects of PPV should be minimized; however, intra-abdominal pressure should be monitored during treatment.

While PPV is associated with certain complications and risks, the procedure generally has a good safety profile. In the present study, all patients showed varying degrees of skin damage; however, there was no reported incidence of pressure ulcers or fatal channel dislodgement. Because PPV should be performed with the assistance of at least 3 to 4 medical staff members, it is extremely important to utilize procedures that will prevent infection of the medical staff. The staff members who assisted with PPV in our study remained non-infected throughout the 35 treatment sessions, which indicated that PPV is safe when performed under strictly enforced protective conditions.

Several limitations of this study need to be acknowledged. First, the study had a small sample size, which might be the reason that only slight non-statistically significant changes were found in most clinical parameters following treatment with PPV. However, as a preliminary investigation, our study explored the impact of PPV on patients with severe H7N9 avian flu accompanied by ARDS. Second, no biomarkers or inflammatory cytokines were examined in this study. It will be important to include these immunology indexes and other clinical parameters in a future systematic study. In conclusion, use of PPV can improve oxygenation in patients with severe H7N9 avian flu accompanied by ARDS, which may decrease CO_2_ retention. Additionally, this improvement is sustained after the patient returns to a supine position. Further studies which include larger patient populations are needed to confirm the hypothesis that application of a respiratory critical care medicine-based combined treatment should significantly reduce the overall mortality rate among such critically ill patients.

## Supporting Information

S1 Table Relevant data underlying the findings described in manuscript. (XLS)
